# Genomic epidemiology of *Staphylococcus aureus* isolated from bloodstream infections in South America during 2019 supports regional surveillance

**DOI:** 10.1099/mgen.0.001020

**Published:** 2023-05-25

**Authors:** Sabrina Di Gregorio, Jesús Vielma, María Sol Haim, Lucía Rago, Josefina Campos, Mihir Kekre, Monica Abrudan, Ángela Famiglietti, Liliana Fernandez Canigia, Gabriela Rubinstein, Martha Helena von Specht, Melina Herrera, Carolina Aro, Marcelo Galas, Norah Balderrama Yarhui, Agnes Figueiredo, Nilton Lincopan, Miryan Falcon, Rosa Guillén, Teresa Camou, Gustavo Varela, David M. Aanensen, Silvia Argimón, Marta Mollerach, on behalf of StaphNET-SA consortium

**Affiliations:** ^1^​ Instituto de Investigaciones en Bacteriología y Virología Molecular (IBaViM), Facultad de Farmacia y Bioquímica, Universidad de Buenos Aires, Ciudad Autónoma de Buenos Aires, Argentina; ^2^​ Consejo Nacional de Investigaciones Científicas y Técnicas (CONICET), Ciudad Autónoma de Buenos Aires, Argentina; ^3^​ Unidad Operativa Centro Nacional de Genómica y Bioinformática, ANLIS Dr. Carlos G. Malbrán, Ciudad Autónoma de Buenos Aires, Argentina; ^4^​ Centre for Genomic Pathogen Surveillance, Big Data Institute, University of Oxford, Oxford, UK; ^5^​ Laboratorio de Bacteriología Clínica, Hospital de Clínicas José de San Martín, Facultad de Farmacia y Bioquímica, Universidad de Buenos Aires, Ciudad Autónoma de Buenos Aires, Argentina; ^6^​ Hospital Alemán, Ciudad Autónoma de Buenos Aires, Argentina; ^7^​ Hospital Privado Regional del Sur, Bariloche, Argentina; ^8^​ Cátedra de Microbiología, Facultad de Ciencias Exactas, Químicas y Naturales, Universidad Nacional de Misiones, Posadas, Argentina; ^9^​ Facultad de Ciencias de la Salud, Universidad Adventista del Plata, Entre Ríos, Argentina; ^10^​ Hospital de Niños Dr. Orlando Alassia, Santa Fé, Argentina; ^11^​ Pan American Health Organization, Washington, DC, USA; ^12^​ Hospital del Niño Manuen Ascencio Villarroel, Cochabamba, Bolivia; ^13^​ Instituto de Microbiologia Paulo de Góes, Universidade Federal do Rio de Janeiro, Rio de Janeiro, Brazil; ^14^​ Programa de Pós-Graduação em Patologia, Faculdade de Medicina, Universidade Federal Fluminense, Niterói, Brazil; ^15^​ Institute of Biomedical Sciences, Department of Microbiology, Universidade de São Paulo, Sao Pablo, Brazil; ^16^​ Dpto. Bacteriología y Micología, Sección Antimicrobianos, Laboratorio Central de Salud Pública, Asunción, Paraguay; ^17^​ Instituto de Investigaciones en Ciencias de la Salud, Facultad de Ciencias Químicas, Universidad Nacional de Asunción, Asunción, Paraguay; ^18^​ Unidad de Bacteriología, Departamento de Laboratorios de Salud Pública, Ministerio de Salud Pública, Montevideo, Uruguay; ^19^​ Cátedra de Bacteriología y Virología, Facultad de Medicina, Universidad de la República, Montevideo, Uruguay; ^20^​ Participating centres, South America; ^†^​ Present address: Tropic Biosciences Ltd, Norwich Research Park, Norwich, UK; ^‡^​ Present address: Ministerio de Salud Pública y Bienestar Social, Asunción, Paraguay

**Keywords:** *S. aureus*, MRSA, MSSA, South America, CC398, CC30, CC5, CC8

## Abstract

*
Staphylococcus aureus* remains one of the leading causes of infections worldwide and a common cause of bacteraemia. However, studies documenting the epidemiology of *S. aureus* in South America using genomics are scarce. We hereby report on the largest genomic epidemiology study to date of both methicillin-resistant *S. aureus* (MRSA) and methicillin-susceptible *S. aureus* (MSSA) in South America, conducted by the StaphNET-SA network. We characterised 404 genomes recovered from a prospective observational study of *S. aureus* bacteraemia in 58 hospitals from Argentina, Bolivia, Brazil, Paraguay and Uruguay between April and October 2019. We show that a minority of *S. aureus* isolates are phenotypically multi-drug resistant (5.2%), but more than a quarter are resistant to macrolide–lincosamide–streptogramin B (MLSb). MSSA were more genetically diverse than MRSA. Lower rates of associated antimicrobial resistance in community-associated(CA)-MRSA versus hospital-associated (HA)-MRSA were found in association with three *S. aureus* genotypes dominating the MRSA population: CC30-MRSA-IVc-*t019-lukS/F-PV+*, CC5-MRSA-IV-*t002-lukS/F-PV-* and CC8-MRSA-IVc-*t008-lukS/F-PV+*-COMER+. These are historically from a CA origin, carry on average fewer antimicrobial resistance determinants, and often lack key virulence genes. Surprisingly, CC398-MSSA-*t1451-lukS/F-PV-* related to the CC398 human-associated lineage is widely disseminated throughout the region, and is described here for the first time as the most prevalent MSSA lineage in South America. Moreover, CC398 strains carrying *ermT* (largely responsible for the MLSb resistance rates of MSSA strains: inducible iMLSb phenotype) and *sh_fabI* (related to triclosan resistance) were recovered from both CA and HA origin. The frequency of MRSA and MSSA lineages differed between countries but the most prevalent *S. aureus* genotypes are high-risk clones widely distributed in the South American region without a clear country-specific phylogeographical structure. Therefore, our findings underline the need for continuous genomic surveillance by regional networks such as StaphNET-SA. This article contains data hosted by Microreact.

## Data Summary

All supporting data, code and protocols have been provided within the article or through supplementary data files. Five supplementary figures and six supplementary tables are available with the online version of this article. Sequence read files for all samples used in this study have been deposited in the European Nucleotide Archive under the project accession number PRJEB37318. Individual accession numbers for each sample are also detailed in a microreact project: https://microreact.org/project/staphnet-sa-1st-survey. Genome assemblies are available via Pathogenwatch: https://pathogen.watch/collection/jz7rcy1zv0sk-staphnet-sa-first-survey.

**Impact Statement**

*
Staphylococcus aureus* is a common cause of bacteraemia, a serious life-threatening disease, and the second leading pathogen for deaths associated with resistance in 2019. However, genomic surveillance of *S. aureus* causing invasive infections in South America is limited. Previous surveillance studies have focused on the dissemination of methicillin-resistant *S. aureus* (MRSA) with increasing antimicrobial resistance and/or virulence, but have not characterised methicillin-susceptible *S. aureus* (MSSA) in detail. Here, we show the results of a prospective observational study of genomic surveillance of *S. aureus* causing bacteraemia conducted in South America during 2019 by the StaphNET-SA network. Our study reveals that in 2019 most bloodstream infections were caused by successful MRSA lineages of community origin, generally not multi-drug resistant, and lacking key virulence genes in some cases. Importantly, we also describe here for the first time CC398-MSSA-*t1451* as the most prevalent and widely disseminated MSSA clone causing bacteraemia in the region during 2019. This human-adapted clone, present in both the community and the hospital environment, carries a gene conferring resistance against an antiseptic widely used in our region, and is largely responsible for the increasing resistance rates to erythromycin and clindamycin observed in MSSA. We also show evidence of ready transmission of the most prevalent MRSA and MSSA high-risk clones across country borders, which highlights the need for continuous genomic surveillance by regional networks such as StaphNET-SA.

## Introduction

*Staphylococcus aureus* causes multiple types of pathologies ranging from moderately severe skin infections, food poisoning, to fatal pneumonia and sepsis. Worldwide, it is one of the most frequently isolated microorganisms in both nosocomial and community-acquired infections associated with high morbidity and mortality [1] and the second leading pathogen for deaths associated with resistance in 2019 [2].

The emergence and dissemination of methicillin-resistant *S. aureus* (MRSA) with additional resistance to other antimicrobial agents is a serious problem both in the hospital (HA-MRSA) and the community (CA-MRSA) environment [3]. Nevertheless, the distinction between community and hospital strains has been blurred and CA-MRSA and HA-MRSA strains have been described as causing outbreaks in both settings [4, 5].

The success of *S. aureus* as a human pathogen relies not only on its ability to develop resistance to most antimicrobial agents introduced into clinical practice, but also on its capacity to produce a diverse set of virulence determinants, including components that enable host colonization and a variety of toxins and immune evasion factors. Different epidemic *S. aureus* lineages represent a serious threat to public health around the world. These high-risk clones (HRCs) may combine greater virulence or transmission potential with resistance to multiple antimicrobial families [6], and their prevalence usually changes over time and with geography.

This scenario is also true in South America, where MRSA causes around 50 % of all *S. aureus* infections in most countries [7]. A regional study performed between 2011 and 2014 revealed that the main MRSA clones causing bacteraemia belonged to the CC5, CC8 and CC30 clonal complexes [8]. ST5-MRSA-I (Cordobes/Chilean clone) and ST105-MRSA-II replaced ST239-MRSA-III (Brazilian clone) as the prevalent HA-MRSA epidemic clones. Meanwhile, ST30-MRSA-IV and ST5-MRSA-IV were the main genotypes associated with CA-MRSA in the southern cone of the region [8, 9]. In contrast, ST8-MRSA-IV, related to the hypervirulent clone USA300, also known as USA300 Latin American variant or South American Epidemic (USA300-LV or USA300-SAE), was found to be predominant in Colombia, Ecuador and Venezuela [8, 10].

Traditionally, surveillance programmes focus on MRSA, but efforts are needed to understand the epidemiology of MSSA infections, which are also of high burden, are increasing in prevalence [11–13] and can give rise to MRSA. Clonal replacement appears to be a common phenomenon, and continuous surveillance is crucial to identify changes in the molecular epidemiology of both MRSA and MSSA. Genomic epidemiology using whole-genome sequencing (WGS) is a powerful tool for surveillance programmes and can provide valuable information on the emergence of HRCs, antibiotic resistance mechanisms and virulence determinants [14]. However, studies documenting the epidemiology of *S. aureus* in Latin America using genomics are scarce [8–10, 15].

We have established a network for collaborative surveillance that can characterise the geographical and temporal dynamics of MSSA and MRSA and their epidemic patterns in South American countries (StaphNET-SA network). The aim of this study was to describe the circulating *S. aureus* clones in the region during 2019 using WGS, to compare them with the global population, and to develop regional capabilities for the sequencing and bioinformatic analysis of whole genomes of *S. aureus* in South America.

## Methods

### Bacterial isolates and study design

A prospective observational study of genomic surveillance of *S. aureus* bacteraemia was conducted in 58 participating hospitals from Argentina, Bolivia, Brazil, Paraguay and Uruguay during 2019. Each participating centre aimed to collect the first five successive MSSA and the first five successive MRSA primary isolates (always from different individuals) obtained from blood cultures between April and October 2019. When a hospital was unable to collect five MRSA or MSSA isolates within the sampling period, the quota of ten could be reached by submitting additional MSSA or MRSA isolates, respectively (Table 1, Fig. S1, available in the online version of this article). This study protocol was previously employed for two European structured surveys of *S. aureus* [16, 17].

**Table 1.** Summary of collected isolates by country CA, community associated; HA, hospital associated; nd, data not available. Totals are given in bold type.

Country	Collected strains	Hospitals (*n*)	MRSA			Total MRSA	MSSA			Total MSSA	Total
			CA	HA	**nd**		CA	HA	**nd**		
Argentina	10 (5 MRSA/5 MSSA)	9	25	20	0	45	17	28	0	45	**90**
	10 (≠5/5)	7	11	15	1	27	17	26	0	43	**70**
	<10	11	14	6	0	20	23	25	1	49	**69**
	Total	**27**	**50**	**41**	**1**	**92**	**57**	**79**	**1**	**137**	**229**
Bolivia	10 (5 MRSA/5 MSSA)	4	9	11	0	20	14	6	0	20	**40**
	10 (≠5/5)	1	3	0	0	3	5	2	0	7	**10**
	<10	4	6	2	0	8	5	2	0	7	**15**
	Total	**9**	**18**	**13**	**0**	**31**	**24**	**10**	**0**	**34**	**65**
Brazil	10 (5 MRSA/5 MSSA)	2	4	6	0	10	3	7	0	10	**20**
	10 (≠5/5)	1	0	0	1	1	8	0	1	9	**10**
	<10	2	1	0	5	6	0	0	5	5	**11**
	Total	**5**	**5**	**6**	**6**	**17**	**11**	**7**	**6**	**24**	**41**
Paraguay	10 (5 MRSA/5 MSSA)	4	9	11	0	20	9	9	2	20	**40**
	10 (≠5/5)	1	5	0	1	6	2	1	1	4	**10**
	<10	4	4	1	0	5	8	1	2	11	**16**
	Total	**9**	**18**	**12**	**1**	**31**	**19**	**11**	**5**	**35**	**66**
Uruguay	10 (5 MRSA/5 MSSA)	1	2	3	0	5	2	3	0	5	**10**
	<10	7	3	3	0	6	10	16	0	26	**32**
	Total	**8**	**5**	**6**	**0**	**11**	**12**	**19**	**0**	**31**	**42**
Total		**58**	**96**	**78**	**8**	**182**	**123**	**126**	**12**	**261**	**443**

Totals are given in bold type.

CA, community associated; HA, hospital associated; ND, data not available.

*S. aureus* identification and antibiotic susceptibility testing (AST) were done in each centre by conventional methods (biochemical tests, disc diffusion, Vitek2, Phoenix and/or Microscan). Antibiotics tested included: oxacillin (OXA), cefoxitin (FOX), erythromycin (ERY), clindamycin (CLI), gentamicin (GEN), ciprofloxacin (CIP), levofloxacin (LVX), rifampin (RIF), trimethoprim-sulfamethoxazole (SXT), minocycline (MNO), vancomycin (VAN) and linezolid (LZD). AST (interpreted as per CLSI 2019 guidelines [18]), origin of infection [community associated (CA) or hospital associated (HA), as per GLASS 2017 definitions [19]], and other relevant demographic and epidemiological metadata (patient age and sex, date of isolation, geographical location, and source of bacteraemia) were collected for each isolate using Epicollect5 (https://five.epicollect.net/) [20]. According to GLASS, the origin of infection was defined as HA origin when the patient’s first isolate was collected on hospital day 3 or later (day of hospital admission is day 1), or CA origin when the patient’s first isolate was collected in the community or on either of the first 2 days of hospitalization. The source of bacteraemia was classified into Skin and soft tissue infection, Respiratory, Catheter, Bone, Surgical wound, Endovascular, Other and Data not available, according to clinical records and curated by the metadata working group of the network to harmonize the data from the different hospitals. All isolates were sent to the School of Pharmacy and Biochemistry (FFyB-UBA), where species identification was confirmed by MALDI-TOF-MS. Antibiotic susceptibility was confirmed by the disc diffusion method as per CLSI 2019 guidelines. All *S. aureus* isolates were retested for methicillin susceptibility while susceptibility to other antibiotics was retested only when genomic results were discordant with AST results.

Multiple drug resistance (MDR) was defined as previously described [21] with the following modifications: (i) susceptibility profiles for LVX and/or CIP were used to define fluoroquinolone resistance; and (ii) tetracycline (MNO), glycopeptide (VAN) and oxazolidinone (LZD) families were not included in the MDR definition due to the high number of isolates not tested for those antibiotics.

### Whole genome sequencing

Genomic DNA was extracted using the Qiacube system (Qiagen), with the addition of lysostaphin(Sigma). DNA was quantified with the Quantus Fluorometer (Promega). WGS was performed at the Wellcome Sanger Institute on the Illumina HiSeq platform with 150 bp paired-end reads.

### Quality control, assembly and annotation

Genomes were quality controlled and assembled from short-read data with the GHRU-AMR pipeline [22], which is described in detail at: https://www.protocols.io/view/ghru-genomic-surveillance-of-antimicrobial-resista-bpn6mmhe. Default parameters were used for all software unless otherwise specified. Genomes were excluded from the study if more than 5 % of the reads belonged to another species, or based on the quality of their assemblies if they contained more than 400 contigs, more than 10 000 ambiguous bases (Ns), their N50 was <14 000, their total length was at least 10 % smaller than the smallest genome or at least 10 % larger than the largest complete *S. aureus* genome in RefSeq, or their GC content was smaller than 32.4 % or larger than 35.1 % (based on the complete *S. aureus* genomes in RefSeq).

### *In silico* genotyping

The *spa* types were derived from assemblies using spaTyper (http://spatyper.fortinbras.us). Multi-locus sequence type (MLST) was determined from reads using the GHRU-AMR pipeline [22]. We used multiple approaches to determine the SCC*mec* type. First, we mapped the sequence reads to an SCC*mec* database [http://www.sccmec.org] of genes defining the *ccr* complex, the *mec* complex and the J1 region [23] with ARIBA v2.14.6 [24], and determined the type and subtype based on the matches and Kondo typing scheme [23]. We complemented the SCC*mec* database with the genes from types XII and XIII. We also analysed the genomes with Staphopia [25], which yielded concordant results for 354/404 genomes. Assemblies of the remaining 50 genomes were analysed with SCCmecFinder [26], which determined a type/subtype for 39 additional genomes. The SCC*mec* cassettes of 11 genomes were non-typable.

### Detection of antimicrobial resistance and virulence determinants, and mobile genetic elements

Detection of antimicrobial resistance (AMR) determinants, virulence genes and mobile genetic elements (MGEs) was carried out with ARIBA v2.14.6 [24] and relevant databases. AMR determinants were determined with the GHRU-AMR pipeline [22], using the NCBI database [27 ] and the pointfinder database (both downloaded 17 February 2021)[28]. Virulence genes were detected using the VFDB database (downloaded 27 August 2021) [29]. Plasmid replicons were detected using the Plasmidfinder database (downloaded 10 September 2021) [30]. Phage integrase types, intSaPI types, insertion sequences and hospital associated (ICEs) were detected as described previously [15].

### Pangenome analysis

Assemblies were annotated with the implementation of Prokka [31] in Panaroo v1.2.0 [32], which was used to determine the pangenome of 404 isolates. SNPs were identified from the resulting core-genome alignment with snp-sites v2.5.1 [33] and were used to build a maximum-likelihood (ML) phylogenetic tree with RAxML v8.2.8 [34] using the GTR+GAMMA model and 500 bootstrap replicates. Pairwise SNP differences were calculated with pairsnp v0.0.7 [35].

### Global context, variant detection and phylogenetic analysis

Genomes belonging to the most prevalent clonal complexes in the study (CC30, CC5, CC8 and CC398) were contextualized with global public genomes (Table S1).

Short paired-end reads were simulated from assemblies for genomes without short-read data available. Contigs smaller than 1 kb were removed from the assemblies with seqkit v0.10.1(0.10.1–−1) [36], and 100 bp paired-end reads were simulated with depth 50× and insert length 500 using pIRS v2.0.2 (2.0.2--pl5.22.0_1) [37].

Reads were then mapped to the corresponding reference genomes (Table S1) using the GHRU snp_phylogeny pipeline [22]. SNPs were identified with snp-sites v2.5.1 [33] from the resulting whole-genome alignments, after excluding regions of MGEs [38 ] and recombination with Gubbins v3.0.0 [39], and were used to build an ML phylogenetic tree with IQ-Tree v1.6.10 [40], with ModelFinder to determine the best-fit model [41 ]. Branch support was estimated with the SH-aLRT test and ultrafast bootstrap (1000 replicates each) [42]. The resulting tree was rooted using an outgroup genome (Table S1) that was omitted from the figures. AMR and virulence determinants, SCC*mec* type, and MGEs were detected for these global collections of genomes with the same methods as described above.

The Microreact web application [43] was used for the integrated visualization of phylogenetic trees, geographical and temporal data, and other associated epidemiological and genetic data.

### Statistical methods

Probability (*P*) values were calculated by Fisher’s exact test for categorical variables, and by Pearson’s chi-squared est for estimating differences in MSSA:MRSA proportions using R [44]. The Wilcoxon rank-sum test, Cohen’s d and Cliff’s delta values were used to estimate differences in pairwise SNP distances between genomes of the same or different country using R and the effsize package [44, 45]. A *P*-value of <0.05 was considered significant in all cases.

Comparison of estimates of diversity were calculated for the MRSA and the MSSA subpopulations in our study. The operational taxonomic unit (OTU) was the sequence type (ST) and novel STs were considered distinct OTUs. Observed and asymptotic estimates of diversity (OTU Richness, Shannon index and Simpson index) and their corresponding 95 % bootstrap confidence intervals were calculated with iNEXT v.2.0.20 [46] with parameters datatype=abundance, se=TRUE, conf=0.95, nboot=50 and endpoint=500.

## Results

### Survey summary

*S. aureus* isolates were collected from blood cultures in 58 hospitals from Argentina, Bolivia, Brazil, Paraguay and Uruguay between April and October 2019. Thirty hospitals reached the objective of ten isolates, out of which 20 hospitals collected the target ratio of 1 : 1 MSSA:MRSA, while the remaining ten hospitals were unable to collect either five MRSA or five MSSA isolates during the sampling period and submitted a different MSSA:MRSA ratio. Twenty-eight hospitals were unable to collect ten isolates (Table 1) . Thus, a total of 443 isolates were collected and the proportion of MRSA and MSSA in our study was 41.1 % (182/443) and 58.9 % (261/443), respectively (Table 1). Notably, MRSA isolates were recovered mainly in the northeast of the sampled region (Fig. S1).

*S. aureus* blood isolates were recovered predominantly from male (287/443, 64.7 %) and adult patients (>18 years) (315/443, 71.1 %). The source of bacteraemia was mainly skin and soft tissue infections (SSTIs) (105/443, 23.7 %) followed by respiratory (86/443, 19.4 %) and catheter (75/443, 16.9 %). Infections with a catheter origin were more frequent in MSSA than in MRSA (Fisher’s exact test, *P*=8.64E-05) (Table 2).

**Table 2.** Infection origin and source of bacteraemia of MRSA and MSSA collected isolates CA, community associated; HA, hospital associated; nd, data not available.

Source of bacteraemia	* S. aureus* (*n,* %)	MRSA			Total MRSA (*n,* %)	MSSA			Total MSSA (*n,* %)
		CA (*n,* %)	HA (*n,* %)	ND (*n,* %)		CA (*n,* %)	HA (*n,* %)	ND (*n,* %)	
Skin and soft tissue infection	105 (23.7 %)	42 (40 %)	12 (11.4 %)	0 (0 %)	54 (51.4 %)	36 (34.3 %)	14 (13.3 %)	1 (1.0 %)	51 (48.6 %)
Respiratory	86 (19.4 %)	21 (24.4 %)	14 (16.3 %)	0 (0 %)	35 (40.7 %)	23 (26.7 %)	26 (30.2 %)	2 (2.3 %)	51 (59.3 %)
Catheter	75 (16.9 %)	3 (4 %)	17 (22.7 %)	0 (0 %)	20 (26.7 %)	10 (13.3 %)	44 (58.7 %)	1 (1.3 %)	55 (73.3 %)
Data not available	54 (12.2 %)	7 (13.0 %)	7 (12.9 %)	6 (11.1 %)	20 (37.0 %)	14 (25.9 %)	13 (24.1 %)	7 (13.0 %)	34 (63.0 %)
Other	41 (9.3 %)	9 (22 %)	7 (17.1 %)	2 (4.9 %)	18(43.9 %)	12 (29.3 %)	10 (24.4 %)	1 (2.4 %)	23 (56.1 %)
Bone	31 (7.0 %)	9 (29.0 %)	4 (12.9 %)	0 (0 %)	13 (41.9 %)	16 (51.6 %)	2 (6.5 %)	0 (0 %)	18 (58.1 %)
Endovascular	30 (6.8 %)	5 (16.7 %)	7 (23.3 %)	0 (0 %)	12 (40 %)	9 (30 %)	9 (30 %)	0 (0 %)	18 (60 %)
Surgical wound	21 (4.7 %)	0 (0 %)	10 (47.6 %)	0 (0 %)	10 (47.6 %)	3 (14.3 %)	8 (38.1 %)	0 (0 %)	11 (52.4 %)
Total	443 (100 %)	96 (21.7 %)	78 (17.6 %)	8 (1.8 %)	182 (41.1 %)	123 (27.8 %)	126 (28.4 %)	12 (2.7 %)	261 (58.9 %)

We determined the resistance rates for 12 antibiotics overall, and for combinations of MRSA or MSSA and HA or CA. The highest resistance rates overall were found for erythromycin (29.7 %) and clindamycin (26.9 %), followed by gentamicin (16.0 %) and fluoroquinolones (ciprofloxacin 12.0 % and levofloxacin 8.5 %) (Table 3). All tested isolates were susceptible to vancomycin (*n*=296) and linezolid (*n*=196).

**Table 3.** Antimicrobial susceptibility of *S. aureus* isolates *N* tested=number of isolates tested for any given antibiotic. Results are expressed as the percentage of tested isolates that are non-susceptible (% Intermediate + % Resistance, %I+R). ERY, erythromycin; CL, clindamycin; GEN, gentamicin; SXT, trimethoprim-sulfamethoxazole; RIF, rifampin; CIP, ciprofloxacin; LVX, levofloxacin; MNO, minocycline.

Antibiotic	* S. aureus *		MRSA, *n*=182		MSSA, *n*=261		*P*-value	MRSA-HA *n*=78		MRSA-CA, *n*=96		*P*-value	MSSA-HA, *n*=126		MSSA-CA, *n*=123		*P*-value
	*N* tested	*N* (%I+R)	*N* tested	*N* (%I+R)	*N* tested	*N* (%I+R)		*N* tested	*N* (%I+R)	*N* tested	*N* (%I+R)		*N* tested	*N* (%I+R)	*N* tested	*N* (%I+R)	
ERY	441	131 (29.7 %)	180	54 (30 %)	261	77 (29.5 %)	0.91	78	36 (46.2 %)	94	16 (17.0 %)	5.10E-05	126	34 (27.0 %)	123	37 (30.0 %)	0.67
CLI	443	119 (26.9 %)	182	43 (23.6 %)	261	76 (29.1 %)	0.23	78	30 (38.5 %)	96	12 (12.5 %)	8.11E-05	126	34 (27.0 %)	123	36 (29.3 %)	0.77
GEN	431	69 (16.0 %)	178	49 (27.5 %)	253	20 (7.9 %)	9.13E-08	77	26 (33.8 %)	93	23 (24.7 %)	0.23	122	9 (7.4 %)	119	9 (7.6 %)	1
SXT	437	5 (1.1 %)	179	5 (2.8 %)	258	0 (0.0 %)	0.01	78	4 (5.1 %)	93	1 (1.1 %)	0.17	124	0 (0.0 %)	122	0 (0.0 %)	1
RIF	428	11 (2.6 %)	172	9 (5.2 %)	256	2(0.8 %)	0.008	77	5 (6.5 %)	87	4 (4.6 %)	0.73	124	1 (0.8 %)	120	1 (0.8 %)	1
CIP	383	46 (12.0 %)	158	29 (18.4 %)	225	17 (7.5 %)	0.002	68	19 (27.9 %)	88	10 (11.4 %)	0.012	113	6 (5.3 %)	106	11 (10.3 %)	0.2
LVX	330	28 (8.5 %)	131	23 (17.5 %)	199	5 (2.5 %)	2.38E-06	60	13 (21.7 %)	64	7 (10.9 %)	0.14	94	2 (2.1 %)	94	3 (3.2 %)	1
MNO	364	2 (0.5 %)	145	1 (0.7 %)	219	1 (0.4 %)	1	67	1 (1.5 %)	76	0 (0.0 %)	0.46	112	1 (0.9 %)	101	0 (0.0 %)	1

However, MRSA strains exhibited higher AMR rates for fluoroquinolones (ciprofloxacin and levofloxacin), rifampin, trimethoprim-sulfamethoxazole and gentamicin than MSSA (Fisher’s exact test, *P<*0.05, Table 3). A comparison of the origin of the infection showed that HA-MRSA resistance rates to ciprofloxacin, erythromycin and clindamycin were significantly higher than those of CA-MRSA (Fisher’s exact test, *P*<0.05, Table 3). No significant differences in resistance rates were found between CA-MSSA and HA-MSSA (Table 3). Nevertheless, differences in these proportions were found between countries, such as Bolivia, where erythromycin and clindamycin resistance is not as prevalent in MSSA (11.8 and 14.7 %, respectively) (Table S2).

### Different populations of MRSA and MSSA in South America

A total of 404 out of 443 collected *S. aureus* isolates in 58 hospitals passed all quality controls (Fig. S2), 239 of which were defined as MSSA and 165 as MRSA based on the absence or presence of the *mecA* gene, respectively.

**Fig. 1.**
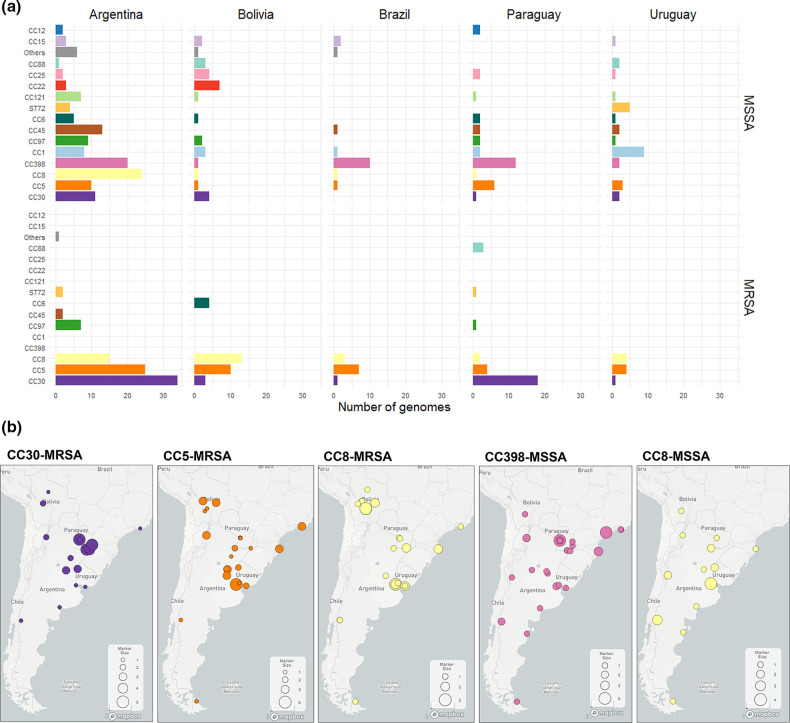
Distribution of clonal complexes (CCs) by country. (**a**) Frequency of CCs by country and MRSA or MSSA. STs comprising fewer than three genomes are grouped under ‘Others’. (**b**) Geographical localization of major CCs in the region. The pie charts on the maps depict the distribution of CCs at each sampling site; the pie size is scaled by the number of genomes collected at each site. Project views are available in microreact: https://microreact.org/project/staphnet-sa-1st-survey.

Among them, we identified 59 different allelic profiles/STs grouped into 14 CCs and nine singletons. The MSSA were more diverse than the MRSA (as measured by the Shannon and Simpson diversity indexes, Table S3), with 44 STs grouped into 14 CCs and eight singletons, and 20 STs grouped into seven CCs and two singletons, respectively. CC30 (18.6 %), CC5 (17.6 %), CC8 (15.8 %) and CC398 (11.1 %) were the most prevalent overall and were recovered from all five countries (Figs 1 and S3). In total, 87 % of the MRSA belonged to three CCs, CC30 (34.5 %), CC5 (30.3 %) and CC8 (22.4 %), while CC398 (18.8 %), CC8 (11.3 %) and CC1 (9.6 %) were the most prevalent CCs among the MSSA population (39.7 %, Figs 1a and S3). Notably, CC30 and CC398 were the most prevalent clones in bacteraemia with a source on SSTIs caused by MRSA and MSSA, respectively (Table S4).

**Fig. 2.**
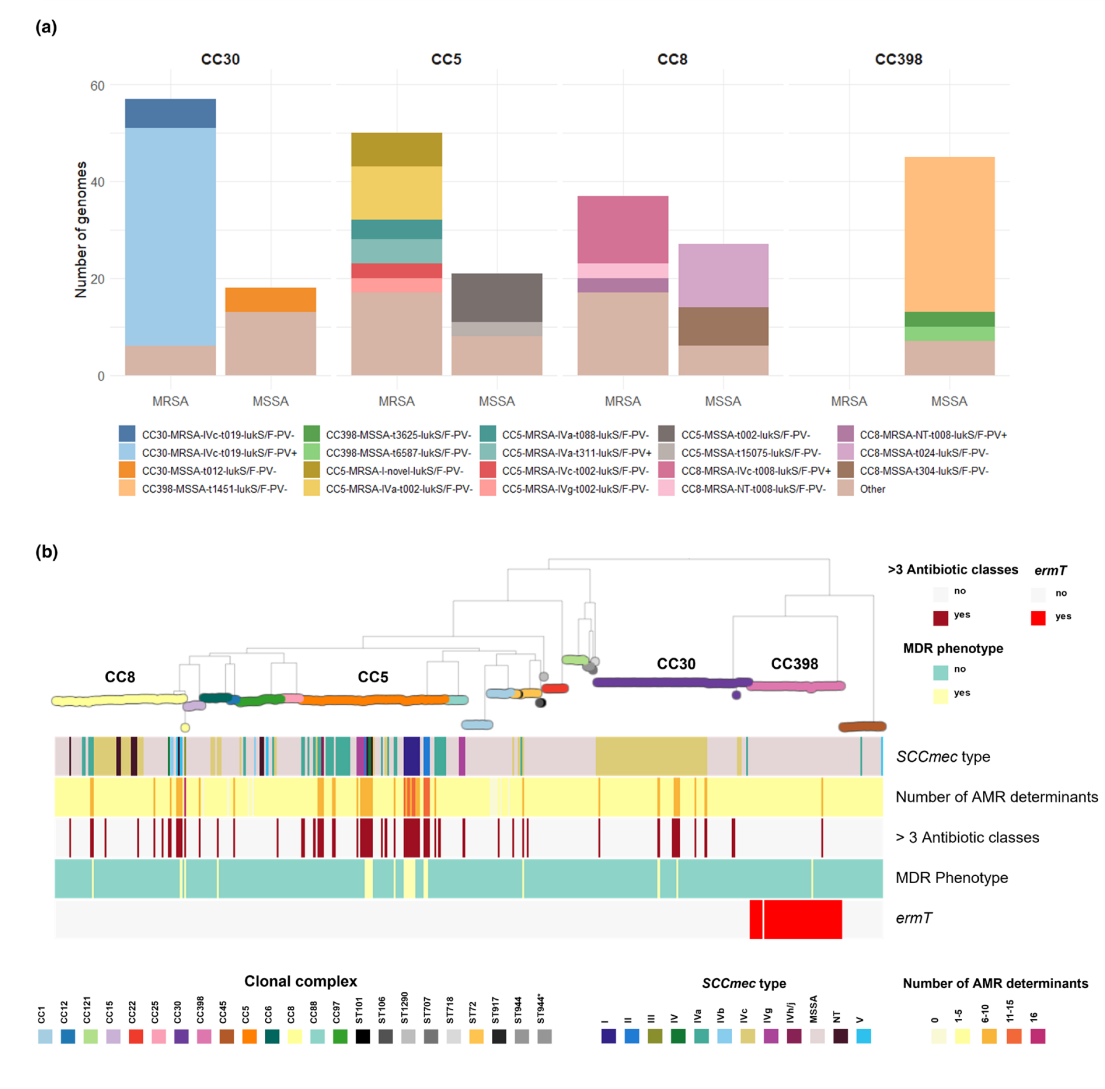
**(a**) Genotypes of the main clonal complexes in the study. ‘Other’ includes genotypes present in fewer than three genomes. (**b**) ML tree of 404 genomes inferred from 156 868 SNP sites identified on 2182 core genes (Panaroo) with RAxML: midpoint rooted; 500 bootstrap replicates. Tree nodes and blocks are coloured as described in the key. MDR phenotype was defined as previously described [21] for all antibiotics tested including: gentamicin, cefoxitin, erythromycin, clindamycin, fluoroquinolones, trimethoprim-sulfamethoxazole, and rifampin. ‘>3 antibiotic classes’: genome with AMR determinants for more than three antibiotic classes including those detailed above plus phenicols, fusidic acid, fosfomycin, mupirocin and bleomycin (not tested phenotypically). Project view is available in microreact: https://microreact.org/project/staphnet-sa-1st-survey.

Moreover, CC398-MSSA is not only described here for the first time as the most prevalent MSSA lineage, but is also widely disseminated throughout the region (Fig. 1b). CC398-MSSA was found to be the dominant MSSA lineage in Brazil and Paraguay, and the second most prevalent in Argentina, where CC8-MSSA is also widely distributed. CC398-MSSA harbours exclusively the *ermT* gene, mainly *spa* type *t1451* and lacks *lukF/S-PV* genes (Fig. 2).

Nonetheless, differences in the frequency of CCs were also found amongst countries, despite few similarities between those sharing borders (Fig. 1). Less prevalent MSSA CCs overall still prevail in Uruguay (CC1-MSSA) and Bolivia (CC22-MSSA). CC30-MRSA remained prevalent in Argentina and Paraguay, consistent with previous reports [47–49], while CC8-MRSA and CC5-MRSA prevailed in Bolivia and Brazil, respectively (Fig. 1a).

The MSSA and MRSA subpopulations of CC30, CC5 and CC8 could be differentiated into genotypes defined by molecular markers traditionally used to describe the epidemiology of *S. aureus *, i.e. the SCC*mec* type, *spa* type and the presence of *lukF/S*-PV genes (Fig. 2). Genotypes CC30-MRSA-IVc-*t019*-*lukF/S*-PV*+*, CC8-MRSA-IVc-*t008*-*lukF/S*-PV+ *and* CC5-MRSA-IV*-t002-lukF/S-*PV*-* (carrying different *SCCmec* IV subtypes) were the most prevalent in the MRSA subpopulation (Fig. 2). Some *spa* types (such as *t311* and *t002)* were found in both MRSA and MSSA within the same CC (e.g. CC5), indicating that the SCC*mec* cassette could have been acquired/lost during the evolution of this CC in the South American cone. However, *spa* types *t019* and *t008,* found only in CC30-MRSA and CC8-MRSA, respectively, have no counterpart in MSSA (Fig. 2a), suggesting that the SCC*mec* cassette was acquired prior to their arrival in the region. Nevertheless, the presence of MSSA-*t019*/*t008* causing infections at low frequency or colonizing individuals should not be disregarded.

In agreement with AST results, *S. aureus* genomes in our collection usually carry a relatively low number of AMR determinants (360/404, 80.1 % carry five or fewer AMR determinants). MDR isolates, i.e. phenotypically resistant to at least three antibiotic classes, were not frequently recovered (21/404, 5.2 %). However, we found 64/404 (15.8 %) genomes with AMR determinants to at least three antibiotic classes, due to the *in silico* detection of resistance to antibiotics not routinely tested, such as fosfomycin (*fosB,* 245/404). Nonetheless, the concordance between genotypic and phenotypic resistance was high (>98.5 %) for eight antibiotics analysed (Table S5, Fig. S4). MDR isolates (21/404) mostly belonged to minor genotypes within CC5, all of them *lukF/S-PV* negative (ST5-MRSA-I-*tnovel*|*others*, ST105-MRSA-II-*t002*|*t985*, ST100-MRSA-NT|IV-*t002*, and ST5-MRSA-II-*t509*) (Fig. 2b). Different genotypes within CC5 contributed to the higher AMR rates in MRSA (fluoroquinolones, gentamicin) and in HA-MRSA (fluoroquinolones and MLSb). CC30-MRSA-IVc*-t019* is also responsible for gentamicin resistance rates while trimethoprim-sulfamethoxazole resistance was linked to CC6-MRSA-IVc-*t701* (Figs 2b and S4).

### Prevalent lineages in a global context

To investigate the evolutionary relationships between the South American MRSA and MSSA, we contextualized genomes belonging to CC30, CC5, CC8 and CC398 with published genomes from different countries and continents, which we describe next.

#### CC30

Several epidemic clones have emerged within CC30, i.e. MSSA-PVL+phage type 80/81 (ST30 [50]), hospital-associated EMRSA-16 (ST36 [51]), MRSA-PVL+South West Pacific (SWP) clone (ST30 [52, 53], and EMSSA-ST30 [6]. A prospective study of *S. aureus* bloodstream infections from nine South American countries showed that MRSA-ST30 was prevalent in Argentina but less so in other countries [8]. The Argentinean MRSA-ST30 isolates were subsequently shown to be diverse, with most isolates belonging to one geographically disseminated clade (ARG-4) related to the SWP clone, but differing from it by their PVL phage type [15].

**Fig. 3.**
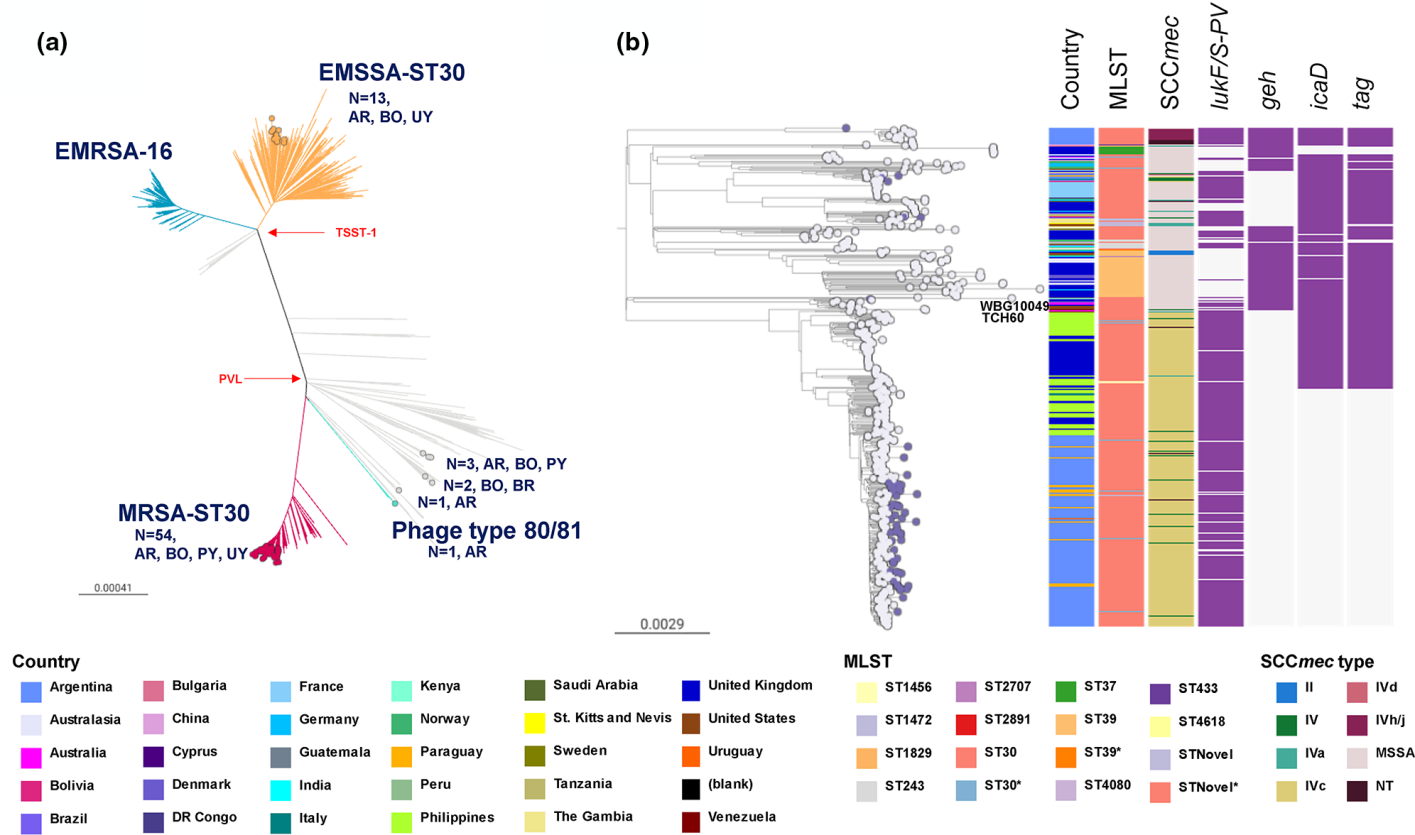
(a) CC30 global phylogeny (the divergent ST34 clade is not represented). Branches are coloured by clade: EMSSA-ST30 (orange), EMRSA-16 (blue), MRSA-ST30 (red), phage type 80/81 (aqua). Coloured nodes represent StaphNET-SA genomes, detailing number and country of origin. AR: Argentina, BO: Bolivia, BR: Brazil, PY: Paraguay, UY: Uruguay. Project is available in microreact: https://microreact.org/project/cc30-global-context. (b) Detailed ST30 phylogeny including 61 genomes from our collection. Outgroup-rooted phylogenetic tree inferred from 61 666 SNP sites obtained after mapping the genomes to the complete genome of strain TCH60 and masking regions of recombination and MGEs. Leaf nodes are coloured by collection: StaphNET-SA (violet), global context (grey). Coloured blocks represent the presence of an intact genetic determinant (purple) or feature as described in the key. For both trees, bars represent the number of SNPs per variable site. Project is available in microreact: https://microreact.org/project/emrsa-st30-global-context.

The CC30 genomes from this study (*n*=75) were mostly found within the MRSA-ST30 (*n*=54) and the EMSSA-ST30 (*n*=13) clones, and closely related to public South American and European genomes. One genome from Argentina and one from Bolivia clustered with the epidemic phage type 80/81 reference genome 55_2053 (Fig. 3a) and within the more divergent ST34, respectively.

As most CC30 genomes from this study (61/75) were found within a clade of 598 genomes supported by a 100 % bootstrap value and including the MRSA-ST30 clone, we inferred a more detailed phylogenetic tree of this group (Fig. 3b). The tree revealed multiple events of acquisition/loss of type IV and type II SCC*mec* cassettes. The notable presence of one monophyletic MRSA clade (100 % bootstrap support) comprising 381 genomes from ten countries with type IVc SCC*mec* was indicative of substantial clonal expansion and global dissemination. The basal branches include two contemporary genomes from Venezuela on a long branch, and a cluster of three genomes from Australia and the USA representing the SWP clone (1999–2004). The isolates from the southern cone of South America, including those from this study (54/75, mainly of CA origin), form a single clade comprising 229 genomes (100 % bootstrap support), consistent with one major introduction followed by clonal spread and geographical dissemination. The basal genomes in the South American clade are from Buenos Aires, Argentina, and genomes from Paraguay (*n*=18), Bolivia (*n*=18), Uruguay (*n*=1) and the UK (*n*=1) are interspersed with genomes from Argentina (*n*=207). Notably, genomes from 2019 (this study) are found on longer branches than those from 2005 to 2014 within this South American clade, and shared *geh*, *icaD* and *tag* mutations already described in the Argentinean genomes [15] (Fig. 3b).

#### CC5

**Fig. 4.**
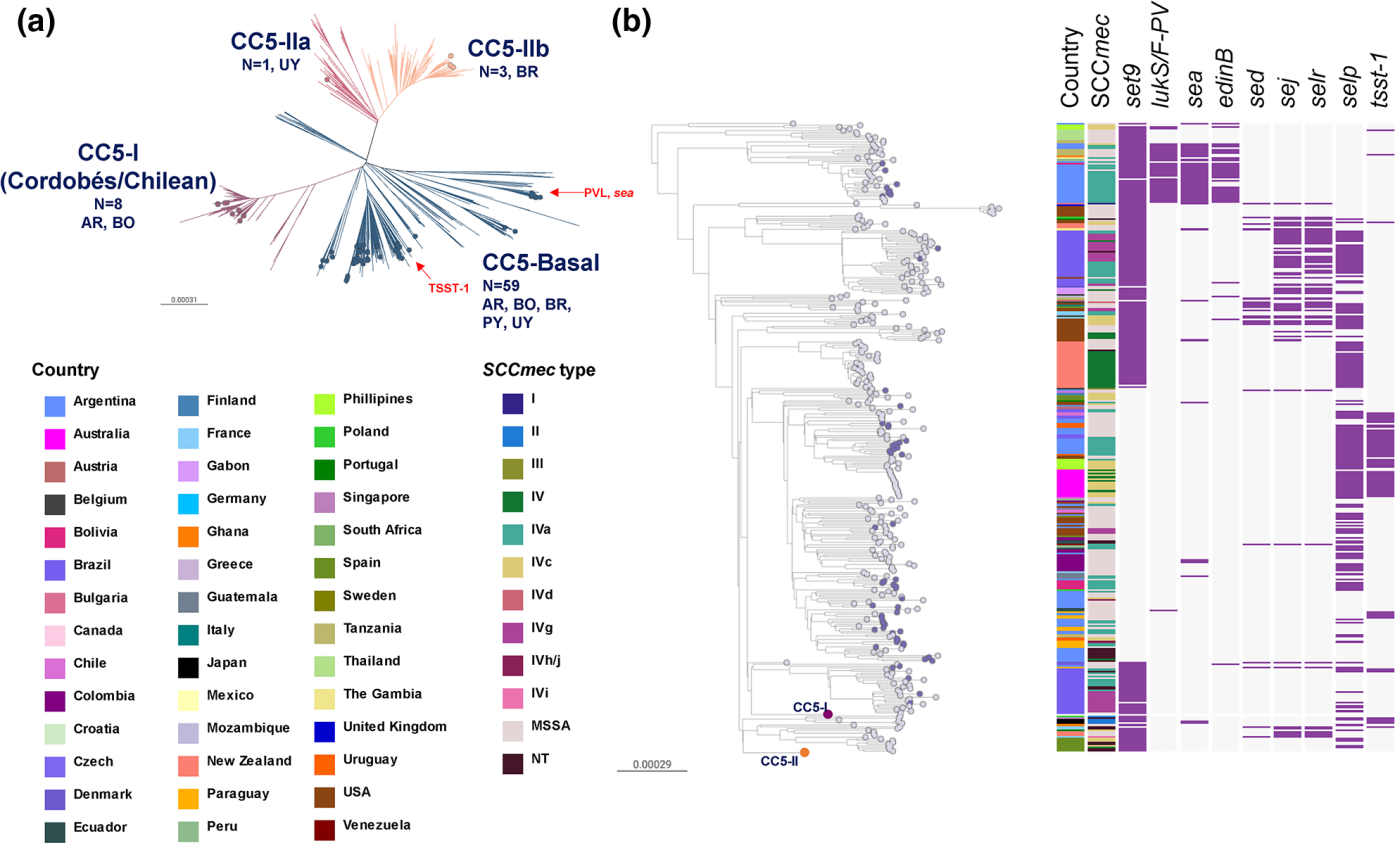
(a) CC5 global phylogeny. Outgroup-rooted phylogenetic tree inferred from 66 837 SNP sites obtained after mapping the genomes to the complete genome of strain JH1 (ST105) and masking regions of recombination and MGEs. Branches are coloured by clade: CC5-Basal (blue), CC5-I (violet), CC5-II-A (light violet), CC5-II-B (salmon). AR: Argentina, BO: Bolivia, BR: Brazil, PY: Paraguay, UY: Uruguay. (**b**) CC5-Basal clades. Same tree from the phylogeny in (a) but CC5-I and CC5-II clades are collapsed as purple (263 genomes) and salmon (436 genomes) circles, respectively. Leaf nodes are coloured by collection: global context (grey), StaphNET-SA (violet). Coloured blocks represent the presence of an intact genetic determinant: virulence gene (violet). Country and *SCCmec* type colours are described in the key. For both trees, bars represent the number of SNPs per variable site. Project is available in microreact: https://microreact.org/project/cc5-global-context.

The diverse set of CC5 genotypes from our collection, predominantly MRSA (50/71; 70.4%) (Fig. 2a), grouped within the four previously defined clades that have caused infections globally [6, 54]: CC5-Basal, CC5-I, CC5-II A and CC5-II B (Fig. 4).

However, the CC5-MRSA lineages once predominant in the region both in the community (ST5-MRSA-IV-*t311* with *lukS/F-PV* and *sea* genes) and in hospitals (CC5-I: ST5-MRSA-I; CC5-II: ST105-MRSA-II and ST5-MRSA-II) [8, 47, 55–57] were scarcely represented and instead replaced by representatives of the CC5-Basal clades (59/71, MSSA or MRSA-IV with *spa* types *t002*, *t311* and related) which are typically not MDR, frequently lack the *set9/ssl4* gene (44/59 genomes) and harbour other toxin genes in a variable manner (Fig. 4b). On the other hand, the persisting members of CC5-I (8/71) and CC5-II (4/71) contribute to CC5, showing the highest AMR rates of any CC in our study (Fig. 2).

Our CC5-Basal genomes are polyphyletic and are interspersed with genomes from South America and also other continents (Fig. 4b), although the global contextualization revealed clades specific to Brazil and Argentina that were not obvious from our data alone (Fig. 4b). Multiple events of acquisition/loss of SCC*mec* IV subtypes (IVa, IVc, IVg, IVh/j) were also evident from the tree. Although CC5-MRSA-IV-*t002* were initially described in the community in Argentina and Brazil [47, 58], they were recovered from both CA (6/59) and HA (10/59) origins in our study. In parallel, we found MDR genomes recovered from CA origin within successful HA-MRSA clades among the less prevalent strains (ST5-MRSA-II and ST105-MRSA-II in Brazil and Uruguay), reinforcing the already described blurred boundaries between the community and the hospital.

#### CC8

**Fig. 5.**
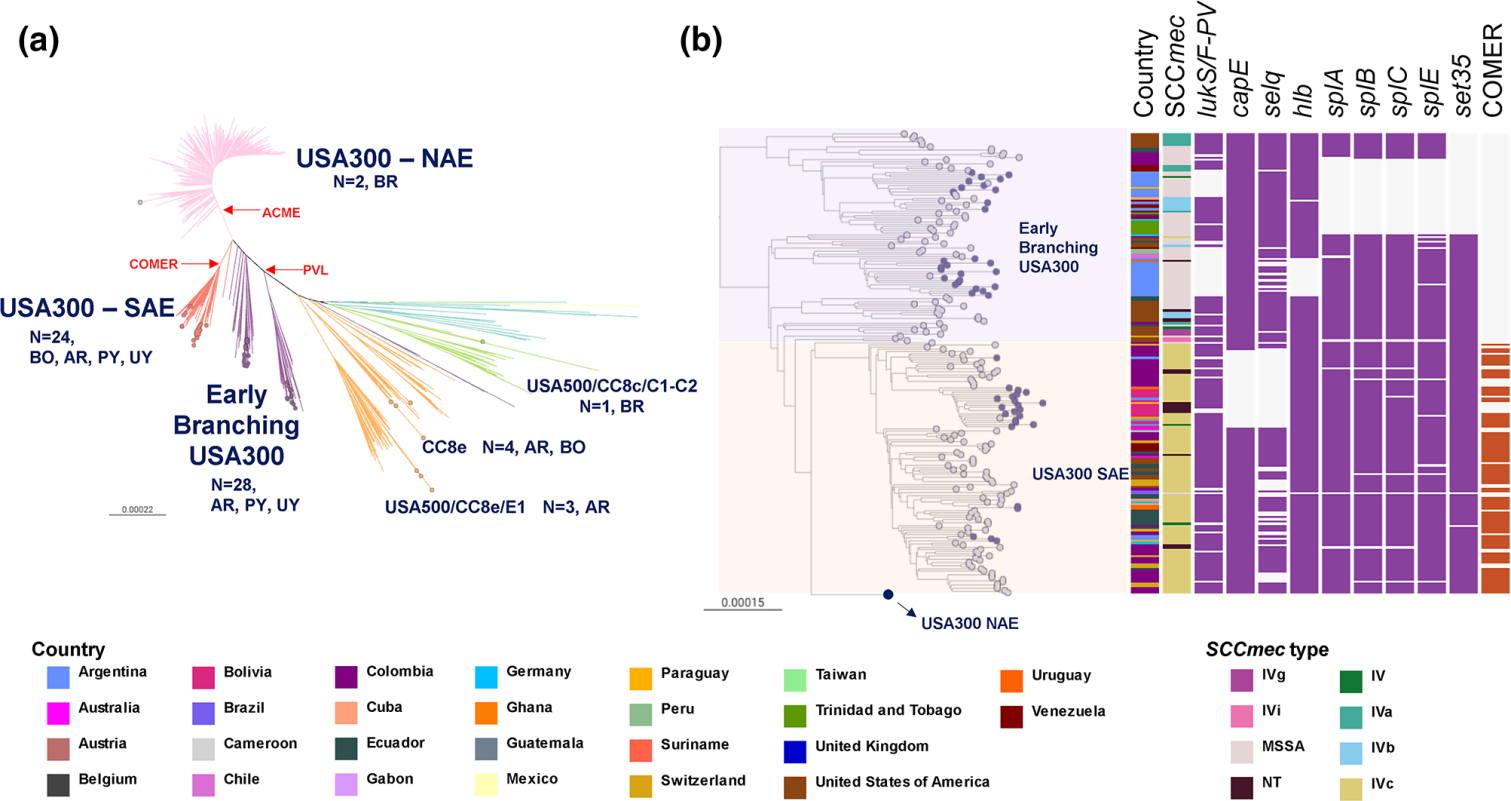
(a) CC8 global phylogeny (more divergent Iberian and ST239 clades are not represented). Outgroup-rooted phylogenetic tree inferred from 66 899 SNP sites obtained after mapping the genomes to the complete genome of strain USA300-FPR3757 (ST8) and masking regions of recombination and MGEs. Coloured nodes represent StaphNET-SA genomes, detailing number and country of origin. AR: Argentina, BO: Bolivia, BR: Brazil, PY: Paraguay, UY: Uruguay. (**b**) Detail of the USA300 subtree from (a). The USA300-NAE clade (760 genomes) is collapsed as a blue circle. Leaf nodes are coloured by collection: StaphNET-SA (violet), global context (grey). Coloured blocks represent the presence of an intact genetic determinant: virulence gene (violet), COMER (brown). Country and SCC*mec* type colours are described in the key. For both trees, the outgroup is omitted, and bars represent the number of SNPs per variable site. Project is available in microreact: https://microreact.org/project/cc8-global-context.

While the relevance of CC8 in South America has been previously linked to the epidemic spread of MRSA lineages (Brazilian clone and USA300-SAE) [10, 55], we found a substantial prevalence of MSSA within this CC (27/64, 42.2%). The majority of our genomes grouped within the previously described USA300-Early Branching (28/64) and USA300-SAE (24/64, MRSA-IVc-*t008-lukF/S-PV*+-COMER+) clades (Fig. 5).

We only found a few genomes related to other CC8 lineages [10, 59, 60] circulating in Brazil (Iberian/CC8b *n*=1, USA500/CC8c *n*=1 and USA300 NAE *n*=2), Bolivia (CC8e *n*=1), and Argentina (CC8e *n*=6 and ST239 Brazilian clone *n*=1).

The USA300-Early Branching genomes were predominantly MSSA widely spread in Argentina (24/28) without a monophyletic origin, suggesting independent introductions (Figs 1 and 5b). One of the early branches comprised 11 genomes of CA origin (7/11) with *spa* type *t304* (8/11) from the centre and north of Argentina (10/11) and Paraguay (1/11) (Fig. S5). The other early branch comprised 17 genomes of HA origin (13/17) with *spa t024* (11/17) in the centre and south of Argentina (16/17) and Uruguay (1/17), showing the sporadic acquisition of *erm*C (Fig. S5). Surprisingly, like related USA300-Early branching genomes from South America (2007–2013), most of our genomes lacked *lukS/F-PV* and other virulence genes (Figs 5b and S5) [10, 59].

The MRSA USA300-SAE lineage known to be prevalent in the north of South America [8, 10] appears to have been introduced into our region on several occasions (Fig. 5b). One such introduction comprised most of our USA300-SAE genomes (18/24), forming a distinct monophyletic group (100 % bootstrap support) widely disseminated across Bolivia (14/18), consistent with the clonal expansion of this lineage in this country and multiple transmission events to Argentina, Paraguay, Uruguay and Switzerland. Of note, the cluster from Bolivia has a clear CA origin (13/18), and lacks functional *selq* and *capE* genes (Fig. 5b).

#### CC398

CC398 was originally described as a livestock-associated lineage, but it is also capable of causing human-to-human transmitted infections as reported from several countries. Phylogenetic evidence later revealed the existence of two distinct CC398 clades (livestock- and human-associated) differing in their repertoire of MGEs and virulence genes [61–65].

**Fig. 6.**
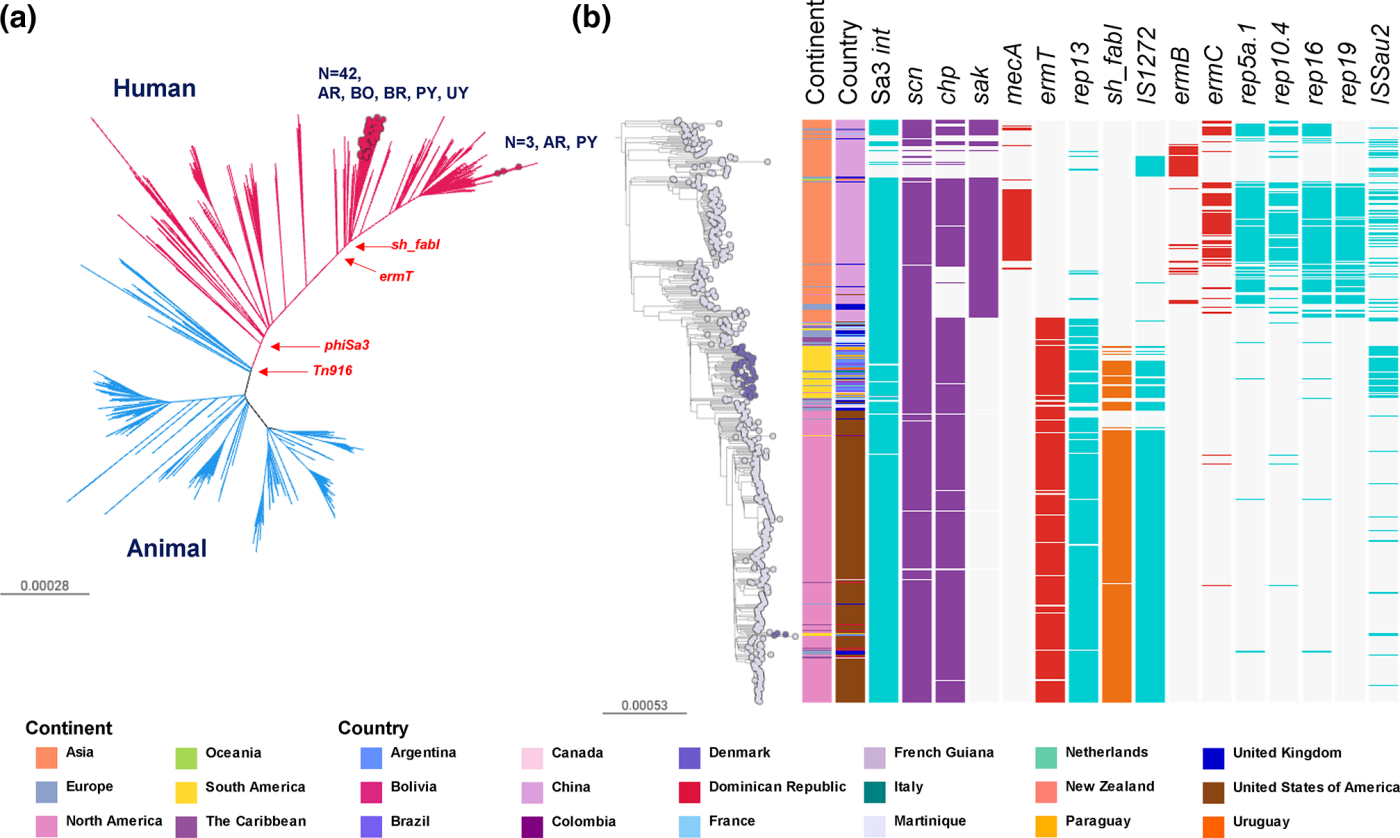
(a) CC398 global phylogeny. Outgroup-rooted phylogenetic tree inferred from 71 097 SNP sites obtained after mapping the genomes to the complete genome of strain S0385 (ST398) and masking regions of recombination and MGEs. Branches are coloured by the host association: animal (blue), human (red). Coloured nodes represent StaphNET-SA genomes, detailing number and country of origin. AR: Argentina, BO: Bolivia, BR: Brazil, PY: Paraguay, UY: Uruguay. (**b**) Human-associated CC398 subtree from (a). Leaf nodes are coloured by collection: StaphNET-SA (violet), global context (grey). Coloured blocks represent the presence of an intact genetic determinant: AMR gene (red), virulence gene (violet), MGE (aqua), triclosan resistance gene (orange). Country and continent colours are described in the key. For both trees, bars represent the number of SNPs per variable site. Project is available in microreact: https://microreact.org/project/cc398-global-context.

The CC398 genomes from this study were recovered from both CA (26/45) and HA (15/45) origin and belonged to *spa* types *t1451* and *t571*, typically linked to human origin (Fig. 1) [65, 66]. Our phylogenetic analysis positioned them within the human-associated clade, closely related to both clinical and carriage isolates (Fig. 6) and lacking *mecA* and Tn*916* (associated with *tetM*) but carrying the *Sa3int* phage-encoded Immune evasion cluster (IEC) genes *chp* and *scn* (but not *sak)* (Fig. 6b).

Nested epidemics could be distinguished within the human-associated clade: one localized in China with occasional transmission to other countries (mainly the UK); a second one in South America involving the majority of the isolates from this study (42/45, from all five countries) clustering as a well-supported group (100 % bootstrap) with ancestral relationships to genomes from the Caribbean, the USA, Europe and China; and a third epidemic mainly localized in the USA that sporadically seeded the Caribbean and South America, including three genomes from this study (two from Argentina and one from Paraguay) (Fig. 6b).

The majority of the genomes in the South American clade carried the *ermT* gene (generally found on the same assembly contig as the *rep13* replicon sequence), the insertion sequences IS*Sau1/*IS*431* and the *cadDX* operon. Manual inspection of assembled genomes suggested that these plasmidic genes have a chromosomal location [65, 67, 68]. We also found IS*1272* and *sh-fabI* (associated with triclosan resistance [69]) in 33/45 genomes. The presence of *ermT, rep13,* IS*1272* and *sh-fabI* was also characteristic of the epidemic in the USA, but not of that in China, where the genomes instead carried *ermB* or *ermC* and other associated plasmidic replicons. On the other hand, an intact copy of IS*Sau2*, prevalent in genomes from our collection and in public genomes from Brazil and Italy found within the South American clade (42/52), was more frequently detected in the genomes from China (60/172) than in those from the USA (10/259) (Fig. 6b).

### Geographical dissemination of major clones

**Fig. 7.**
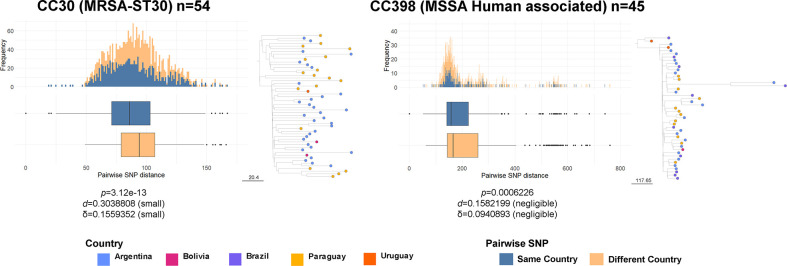
Distributions of core genome SNP differences between pairs of genomes belonging to the same clade from either the same (blue) or different (orange) countries. Pairwise SNP distance is represented as boxplots (median and interquartile range) and a histogram of frequency for each clade. The number of genomes included is detailed in the key for each of the main lineages analysed. Text in the bottom of each figure is an interpretation of the difference between each pair of distributions, obtained using the R package ‘effsize’, which applies the parametric and non-parametric effect size estimators Cohen’s *d* and Cliff’s δ to the results of a Wilcoxon test (*P*). Subtrees of each main clade are represented next to each plot. The bar next to the tree represents the number of SNPs. Nodes (genomes included in the analysis) are coloured by country as described in the key.

To assess the phylogeographical structure of each of the dominant MRSA and MSSA high-risk clones identified in our study, we compared the genetic relatedness between isolates from the same country and from different countries. Pairwise differences in the number of SNPs from core genomes are often used to estimate the level of genetic relatedness between isolates. Comparisons of the pairwise SNP differences within MRSA-ST30 and MSSA-CC398 showed significantly different median pairwise SNP distance between genomes from the same country and genomes from different countries (*P*<0.0001, Wilcoxon test), but the interpretation adjusting for size effects shows that the magnitude of the differences is small or negligible (Cohen’s *d*<0.30, Cliff’s δ<0.16)(Fig. 7). This suggests that, despite their geographical spread, isolates from different countries are, on average, as similar to each other as are isolates from the same country, and further supports the observed frequent transmission across borders of these dominant high-risk clones (Figs 3–6).

## Discussion

Our study provides a contemporary snapshot of the genetic characteristics and the epidemiology of MRSA and MSSA causing bacteraemia in the southern cone of South America during 2019 through WGS and a rational sampling framework like the one used in previous surveys in Europe [16, 17]. However, the sampling framework presented several challenges. We were not always able to recruit enough participating centres to fully represent each country demographically and geographically. The geographical representation of our survey is especially limited in Brazil, where few laboratories were enrolled, and therefore the data probably do not reflect the entire epidemiological situation of this large country. Similarly, the coverage of the southern cone is curtailed by the absence of Chile in this first survey. In addition, almost half of the centres were unable to submit ten *S. aureus* isolates, which may represent both the constraints faced by laboratories in low-resource areas (that nonetheless participated voluntarily), and the decreasing trends in incidence of *S. aureus* bacteraemia [70]. Despite these limitations, our study is the largest genomic survey of the *S. aureus* population in South America to date, which we characterised with a resolution that enabled the identification of MRSA and MSSA lineages of public health significance in the region, informed on their geographical structure, placed them in global context and linked them to AMR phenotypes.

Changes in *S. aureus* AMR trends have been observed in Argentina, Bolivia and Paraguay over the last 8 years [7, 71, 72], probably due to clonal replacements. Our study addresses the gaps in knowledge between phenotypic resistance and population structure by characterising the more prevalent MRSA and MSSA lineages causing bacteraemia occurring in the region during 2019. The higher diversity of MSSA lineages in South America (Fig. 1) parallels similar findings worldwide [16, 73–77]. The most prevalent MSSA genotype (CC398-MSSA-*t1451-lukS/F-PV-*) carrying the *ermT* gene and *sh_fabI* in a MGE associated with triclosan resistance [69] is largely responsible for the erythromycin and clindamycin resistance rates observed in MSSA strains (29.5 and 29.1 %, respectively, Table 3, Fig. 6), which had been rising prior to our sampling period, and continued to rise following the COVID-19 pandemic [71, 72]. To our knowledge, human-associated CC398-MSSA-*t1451-lukS/F-PV*- is described here for the first time as an epidemic MSSA clone in our region.

Between 2006 and 2014, CC398 was only sporadically found in South America colonizing and causing invasive infections in humans [78–83]. Since 2011, in Argentina and Uruguay empirical antimicrobial therapies for SSTIs include clindamycin, trimethoprim-sulfamethoxazole or minocycline due to the high prevalence of CA-MRSA [84, 85 ]. On the other hand, triclosan is an antiseptic widely used in our region in cosmetics at regulated concentrations, but also without regulation in sportswear, toys and other items in contact with human skin[86]. We hypothesize that CC398-MSSA may have recently become a prevalent pathogen in response to the selection pressure imposed by this public health measure, at least in Argentina and Uruguay, or by the indiscriminate use of triclosan, although we cannot rule out other unexplored factors. However, the prevalence of CC398 as an agent of bacteraemia prior to 2019 remains uncertain due to the paucity of surveillance studies of MSSA in South America.

Although cefazolin is a common treatment option for MSSA bacteraemia, older South American bloodstream MSSA isolates carrying BlaZ types A and C have previously exhibited a high frequency of the cefazolin inoculum effect, which is associated with treatment failure and increased mortality [83, 87 ]. Almost half of the MSSA genomes analysed here, including CC398, harboured BlaZ type B (113/239, 47.3 %) (Table S6), which does not lead to this phenotype [87, 88]. However, a high proportion of genomes from other CCs carried BlaZ type A or C (90/239, 37.7 %) (Tale S6), especially in Bolivia (18/31, 58.1 %) where CC398 is not as prevalent. Therefore, although cefazolin seems to be a viable option for the treatment of MSSA in our region, further studies are needed to understand potential country-specific differences in the cefazolin inoculum effect.

Despite the *S. aureus* strains recovered from bacteraemia in our study in general not being MDR (Table 3, Fig. 2), MRSA strains pose an additional challenge to treatment because of higher resistance rates to additional antibiotics besides beta-lactams (Table 3). The MRSA sub-population was dominated by three CCs (CC30, CC5 and CC8), and in particular by three genotypes, CC30-MRSA-IVc-*t019-lukS/F-PV*+, CC5-MRSA-IV*-t002-lukS/F-PV-* and CC8-MRSA-IVc*-t008-lukS/F-PV+*-COMER+ (Fig. 2).

Global phylogenetic analysis of CC30-MRSA-IVc-*t019-lukS/F-PV*+ showed a distinct epidemic of this SWP-related clone affecting the Southern Cone of South America, in particular Paraguay and Argentina, where it is relatively more prevalent than other MRSA. Our findings suggest a single introduction in South America of an ST30-MRSA clone that disseminated throughout the continent, probably via travel routes, in agreement with previous reports [9, 47–49, 85, 89–91]. ST30-MRSA was first described in our region from Uruguay [92] and Brazil [93]. The paucity of genomes from earlier dates precludes us from establishing the entry point into the region with the current analysis.

Interestingly, genomes from this study belonging to CC30-MRSA-IVc-*t019-lukS/F-PV*+ were found on longer terminal branches than related genomes from 2005 to 2014 in the same clade, suggesting the accumulation of more genetic variability (Fig. 3). Our previous work [15] describes the acquisition of a premature stop codon in the *tag* gene (coding for DNA-3-methyladenine glycosylase) ancestral to this clade. The loss of function of this enzyme in charge of removal of chemically damaged DNA might be related to the accumulation of genetic changes observed. *S. aureus* hypermutator strains, caused by defects in the methyl-mismatch repair system via mutations on the *mutSL* locus, have been previously found to play a role in AMR development during long-term persistence [94, 95]. Interestingly, the source of bacteraemia for 25 of 49 CC30-MRSA-IVc-*t019-lukS/F-PV*+ genomes are SSTIs (*n*=22) and surgical wounds (*n*=3), which suggests an ability to cause SSTIs, possibly favoured by long-term colonization of the patient’s skin. In contrast, none of the other CC30-MRSA found on different clades of the CC30 tree (one CC30-MRSA-IVa-*t318-lukS/F-PV+* and two CC30-MRSA-IVc-*t433-lukS/F-PV*+) were known to be from SSTIs or surgical wounds. A possible relationship between DNA repair and the success of this MRSA lineage will be the subject of future investigation (Fig. 3).

The genotype CC5-MRSA-IV*-t002-lukS/F-PV-* was contextualized within the previously defined CC5-Basal clade, as opposed to the CC5 Cordobés/Chilean (or CC5-I) clone once dominant in the region. The polyphyly of CC5-basal genomes was reflected on a variable repertoire of toxins [54] (Fig. 4). Remarkably, most of them lacked the staphylococcal superantigen-like protein *set9*/*ssl4* gene, which is located within the variable region of the genomic island νSaα [96].

We placed CC8-MRSA-IVc-*t008-lukS/F-PV+* COMER+ within the epidemic USA300-SAE clone previously described for the north of South America (Fig. 5) [10]. Importantly, we describe a variant of USA300-SAE lacking a functional capsular biosynthesis gene *capE* as the most prevalent MRSA clone in Bolivia, which probably evolved from Colombian ancestors [10]. Isolates with similar epidemiological markers were reported from human colonization and infections in Bolivia in 2010–2013, suggesting the presence of this clone in the country during the last decade [79, 97]. Our study delivers local value by demonstrating the importance of this clone in Bolivia, as well as its spread to other countries in the southern cone of South America, and link to a public genome from a travel-associated infection in Europe [97].

The lower AMR rates found in CA-MRSA strains compared to HA-MRSA (Fig. 2, Table 3) are linked to two genotypes CC30-MRSA-IVc-*t019-lukS/F-PV*+ and CC8-MRSA-IVc-*t008-lukS/F-PV*+-COMER+ (Fig. 2), which are historically from a CA origin, are overrepresented in CA-MRSA compared to HA-MRSA based on our infection origin definitions, and carry a small number of AMR determinants. Similar to reports from other regions [98–100], MDR HA-MRSA lineages previously dominant in South America (ST239-MRSA-III ‘Brazilian’ clone, ST5-MRSA-I ‘Chilean/Cordobés’ clone, ST105-MRSA-II ‘Rio de Janeiro’ clone) [47, 55, 56, 101, 102] were replaced by non-MDR MRSA that now seem to have fully established within the hospital environment and cause bloodstream infections. The lack of anti-restriction gene homologues previously associated with MDR [103] might partially explain (at least in part) the low levels of AMR in these strains.

In conclusion, the majority of the dominant genotypes causing bacteraemia in South America are HRCs with a CA origin and related to global epidemic clones. Nevertheless, we demonstrate the presence of local and regional epidemics of these HRCs within South America, e.g. of a descendant of the SWP clone (MRSA-ST30) affecting mainly Argentina and Paraguay, of a variant of USA300-SAE mainly in Bolivia, and of CC398-MSSA mainly in Argentina, Paraguay and Brazil. MRSA-ST30 and CC398-MSSA did not exhibit clear country-specific phylogenetic signals (Fig. 7), consistent with ready community transmission amongst colonized individuals in a region where travel between neighbouring countries is frequent.

Several of these clones lacked intact copies of key virulence genes such as *geh*/*icaD*/*tag* in MRSA-ST30, *set9* in CC5-Basal, *lukS*/*F*-PV in USA300-Early branching clades or *capE*/*selq* in USA300-SAE (Figs 3–6), further supporting the added value of genomics in characterising locally circulating lineages and distinguishing them from those found in other regions/countries. This is particularly important in the light of previous work showing that absence/loss of virulence properties and the concomitant reduced toxicity could pave the way for CA clones of *S. aureus* to continue to cause invasive infections in the hospital environment [104–108]. In contrast to virulence gene loss, the sporadic acquisition of AMR determinants we observed within these CA clones could lead to the selection and expansion of new resistant clones. Taking into account previous reports from Brazil showing that CC398-MSSA-*t1451* can acquire the SCC*mec* cassette [58, 80], and the widespread use of antibiotics and antiseptics during the COVID-19 pandemic, the emergence of epidemic CC398-MRSA clones with MLSb resistance is a plausible scenario.

Taken together, our findings highlight the need for regional ongoing genomic surveillance by networks such as StaphNET-SA. This first study of the network focused on isolates recovered from bacteraemia. However, in order to have a full understanding of the impact and burden of MSSA and MRSA on healthcare systems in the region, future genomic surveillance studies will need to include not only isolates recovered from bacteraemia, but from SSTIs and other infection types. Structured surveys undertaken at regular time intervals could uncover the population dynamics of this pathogen through comparison with the baseline population established in this study. We made available the assemblies from this first survey via the PathogenWatch platform to facilitate exploration of these data and comparison with datasets from other countries/regions. Structured surveys have been conducted in Europe in the past [16, 17] and, if adopted in other regions in a standardized and concerted manner, could contribute significantly to a global picture of this pathogen responsible for the highest number of deaths worldwide in 2019 [109].

## Supplementary Data

Supplementary material 1

Supplementary material 2

Supplementary material 3

Supplementary material 4

Supplementary material 5

Supplementary material 6

Supplementary material 7

Supplementary material 8

Supplementary material 9

Supplementary material 10

Supplementary material 11
