# Supplementary material for: Genomic epidemiology of Staphylococcus aureus isolated from bloodstream infections in South America during 2019 supports regional surveillance
Source: Microb Genom. 2023 May 25;9(5):mgen001020. doi: 10.1099/mgen.0.001020 (PMC10272885; doi:10.1099/mgen.0.001020)

Supplementary Figure 1. Sampling map of StaphNET-SA study surveillance sites. Coloured pie charts depict the distribution of MRSA (red) and MSSA (light blue); the pie size is scaled according to the number of isolates collected at each site.

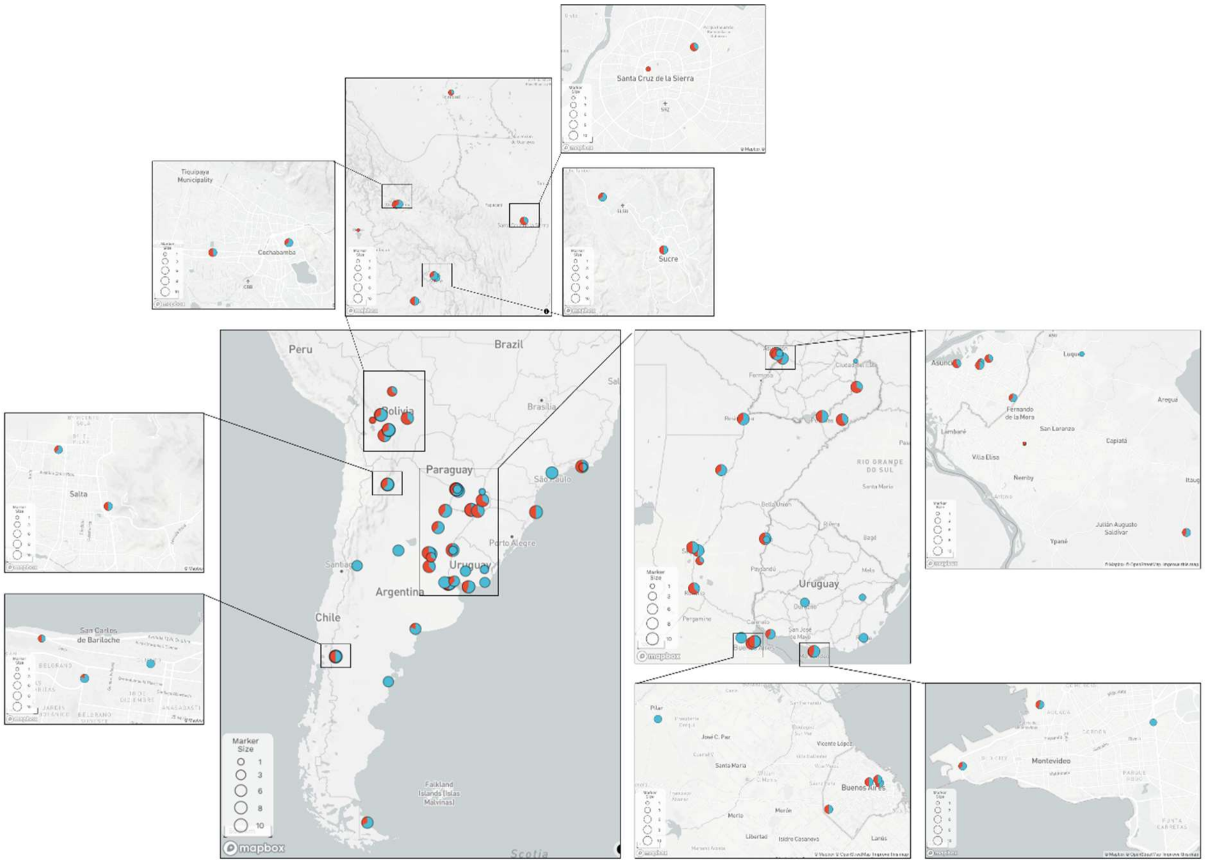

Supplement: Supplementary material 1 [file mgen-9-1020-s001.pdf]
