# Supplementary material for: Genomic epidemiology of Staphylococcus aureus isolated from bloodstream infections in South America during 2019 supports regional surveillance
Source: Microb Genom. 2023 May 25;9(5):mgen001020. doi: 10.1099/mgen.0.001020 (PMC10272885; doi:10.1099/mgen.0.001020)

Supplementary Figure 2. Diagram flow showing confirmation and quality control performed on *S. aureus* isolates and genomes in this study.

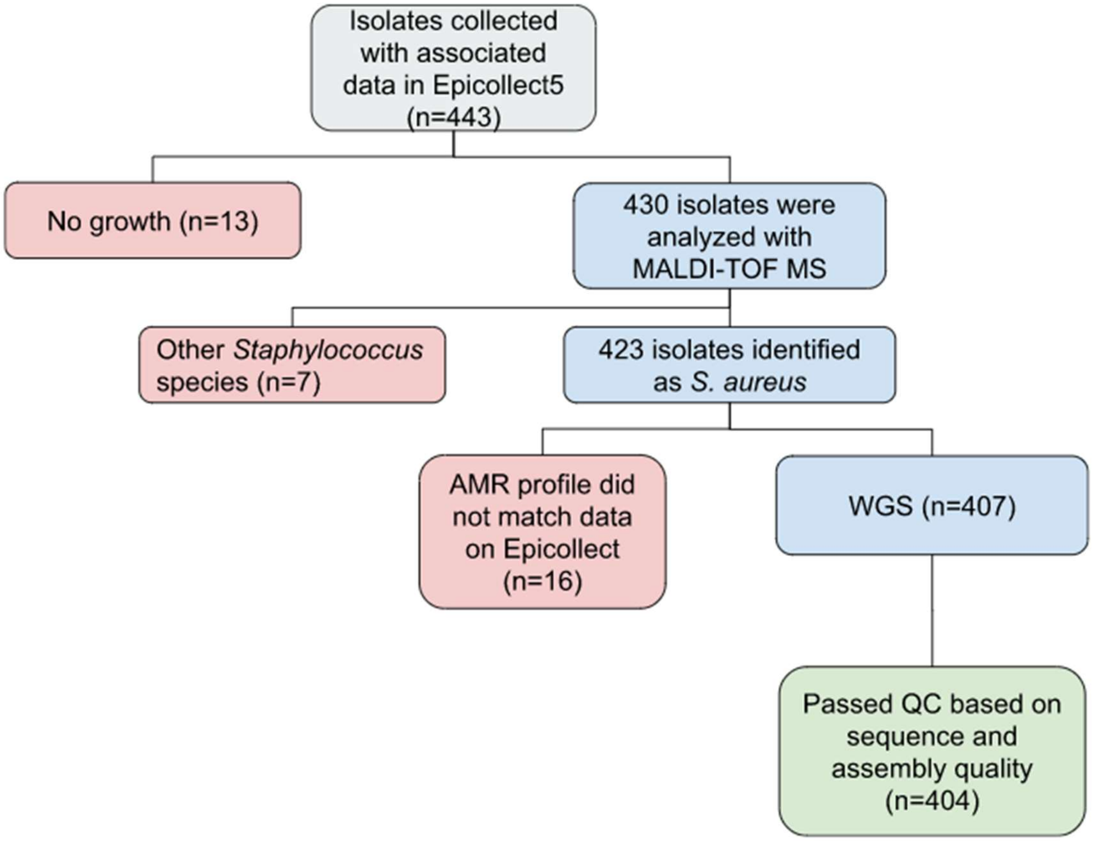

Supplement: Supplementary material 2 [file mgen-9-1020-s002.pdf]
