# Supplementary material for: Genomic epidemiology of Staphylococcus aureus isolated from bloodstream infections in South America during 2019 supports regional surveillance
Source: Microb Genom. 2023 May 25;9(5):mgen001020. doi: 10.1099/mgen.0.001020 (PMC10272885; doi:10.1099/mgen.0.001020)

Supplementary Figure 3. Distribution of clonal complexes in the 404 *S. aureus* genomes by MRSA or MSSA. STs comprising less than 3 genomes are grouped under “Others”. Bars are coloured as described in the legend.

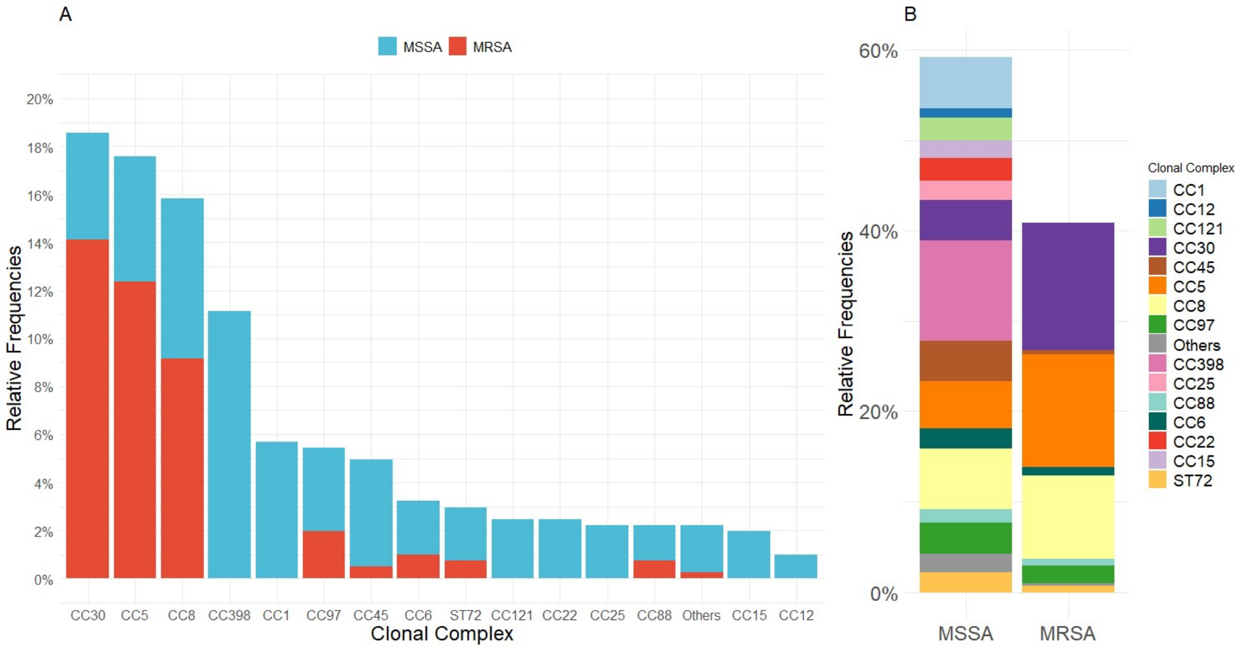

Supplement: Supplementary material 3 [file mgen-9-1020-s003.pdf]
