# Supplementary material for: Genomic epidemiology of Staphylococcus aureus isolated from bloodstream infections in South America during 2019 supports regional surveillance
Source: Microb Genom. 2023 May 25;9(5):mgen001020. doi: 10.1099/mgen.0.001020 (PMC10272885; doi:10.1099/mgen.0.001020)

Supplementary Figure 4. AMR determinants and AMR phenotypes. Maximum Likelihood tree of 404 genomes inferred from 156868 SNP sites identified on 2182 core genes (Panaroo) with RAxML. Midpoint rooted. 500 bootstrap replicates. Tree nodes and blocks are coloured as described in the legend.

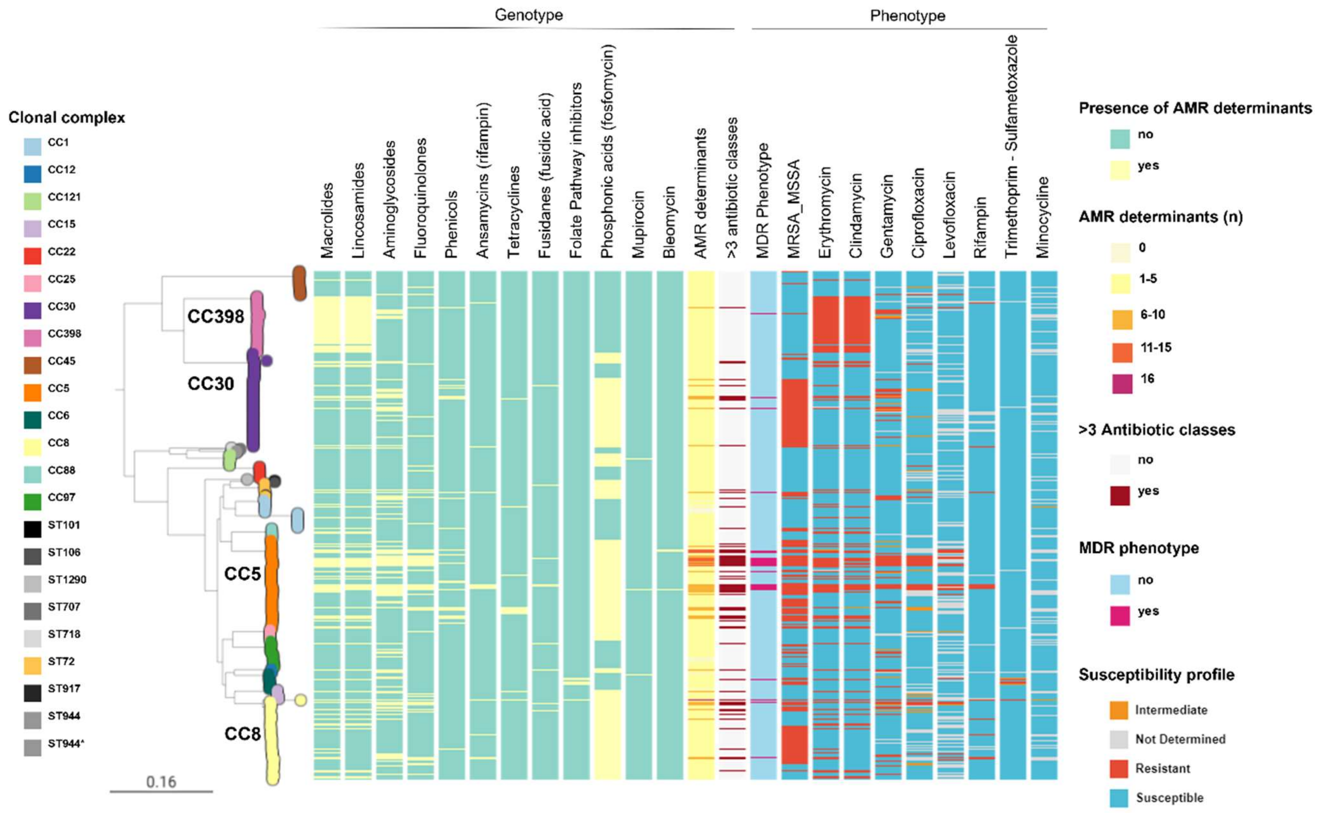

Supplement: Supplementary material 4 [file mgen-9-1020-s004.pdf]
