# Supplementary material for: Genomic epidemiology of Staphylococcus aureus isolated from bloodstream infections in South America during 2019 supports regional surveillance
Source: Microb Genom. 2023 May 25;9(5):mgen001020. doi: 10.1099/mgen.0.001020 (PMC10272885; doi:10.1099/mgen.0.001020)

Supplementary Figure 5. Geographic distribution of USA300 Early Branching clades. Leaf nodes are coloured by Country. Coloured blocks represent the presence of an intact genetic determinant: virulence gene (violet). Country and SCC*mec* type colours are described in the legend. For both trees, the outgroup is omitted, and scale bars represent the number of single nucleotide polymorphisms (SNPs) per variable site.

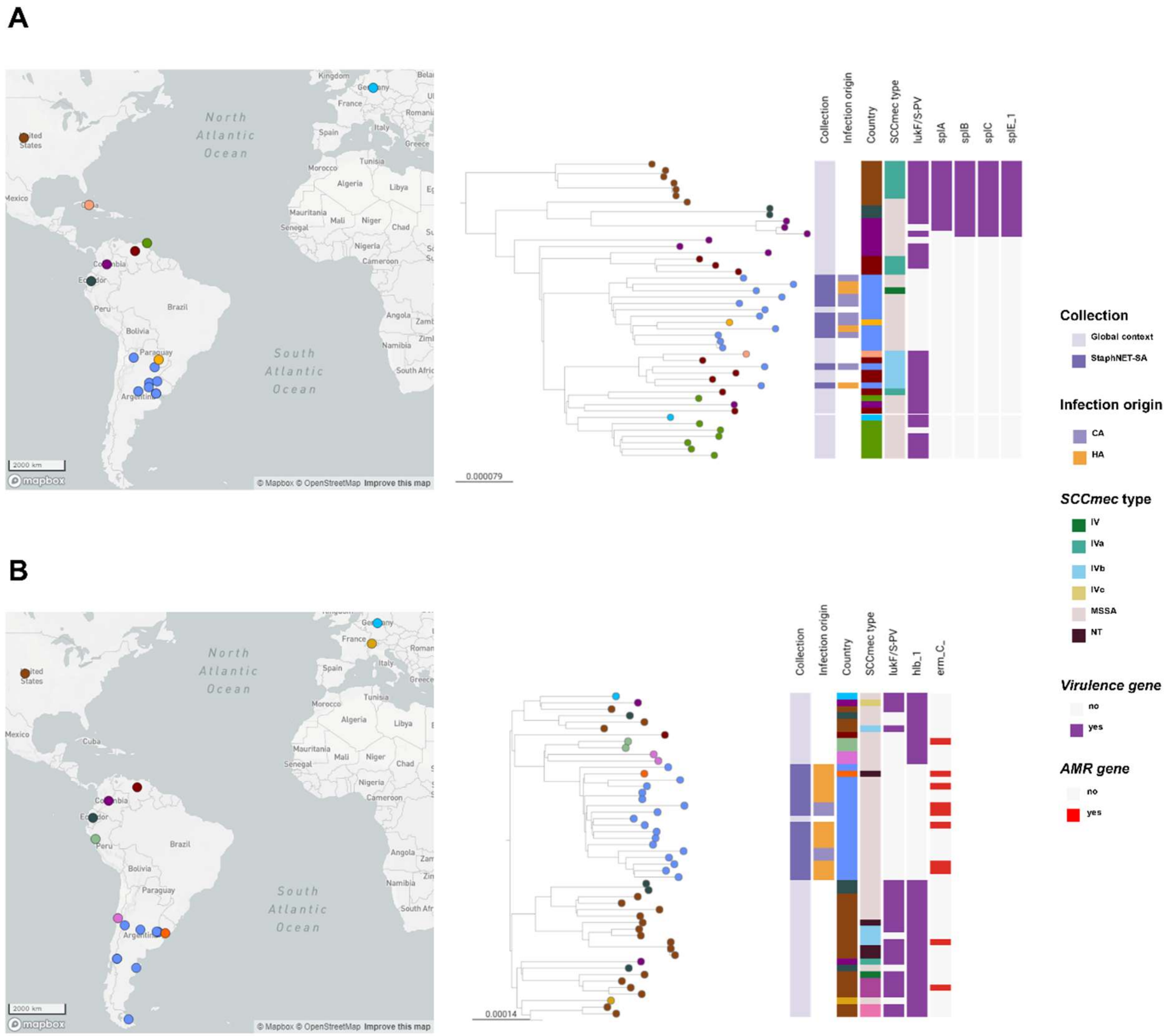

Supplement: Supplementary material 5 [file mgen-9-1020-s005.pdf]
